# Adapting rapid assessment procedures for implementation research using a team-based approach to analysis: a case example of patient quality and safety interventions in the ICU

**DOI:** 10.1186/s13012-020-0972-5

**Published:** 2020-02-22

**Authors:** Laura M. Holdsworth, Nadia Safaeinili, Marcy Winget, Karl A. Lorenz, Mary Lough, Steve Asch, Elizabeth Malcolm

**Affiliations:** 1 Division of Primary Care and Population Health, School of Medicine, Stanford University, 1265 Welch Rd MSOB, Stanford, CA 94305 USA; 2 Veterans Affairs, Palo Alto, CA USA; 3 Stanford Health Care, Palo Alto, CA USA; 4 Division of General Internal Medicine, Duke University School of Medicine, Durham, NC USA

**Keywords:** Qualitative methods, Rapid assessment procedures, Patient safety, Intensive care, Team-based analysis, Hybrid designs

## Abstract

**Background:**

Innovations to improve quality and safety in healthcare are increasingly complex, targeting multiple disciplines and organizational levels, and often requiring significant behavior change by those delivering care. Learning health systems must tackle the crucial task of understanding the implementation and effectiveness of complex interventions, but may be hampered in their efforts by limitations in study design imposed by business-cycle timelines and implementation into fast-paced clinical environments. Rapid assessment procedures are a pragmatic option for producing timely, contextually rich evaluative information about complex interventions implemented into dynamic clinical settings.

**Methods:**

We describe our adaptation of rapid assessment procedures and introduce a rapid team-based analysis process using an example of an evaluation of an intensive care unit (ICU) redesign initiative aimed at improving patient safety in four academic medical centers across the USA. Steps in our approach included (1) iteratively working with stakeholders to develop evaluation questions; (2) integration of implementation science frameworks into field guides and analytic tools; (3) selecting and training a multidisciplinary site visit team; (4) preparation and trust building for 2-day site visits; (5) engaging sites in a participatory approach to data collection; (6) rapid team analysis and triangulation of data sources and methods using a priori charts derived from implementation frameworks; and (7) validation of findings with sites.

**Results:**

We used the rapid assessment approach at each of the four ICU sites to evaluate the implementation of the sites’ innovations. Though the ICU projects all included three common components, they were individually developed to suit the local context and had mixed implementation outcomes. We generated in-depth case summaries describing the overall implementation process for each site; implementation barriers and facilitators for all four sites are presented. One of the site case summaries is presented as an example of findings generated using the method.

**Conclusions:**

A rapid team-based approach to qualitative analysis using charts and team discussion using validation techniques, such as member-checking, can be included as part of rapid assessment procedures. Our work demonstrates the value of including rapid assessment procedures for implementation research when time and resources are limited.

Contributions to the literature

- Rapid assessment procedures have been utilized for studying implementation in health care, but require further development with regards to ensuring quality and rigor among teams of researchers.
- We describe a team-based analysis process utilizing a templated approach derived from implementation science frameworks and using an iterative group process of reviewing and reflecting on the data, sorting and categorizing, and meaning-making using charts.
- This team-based approach, along with a range of validation techniques, such as member checking, can generate credible, trustworthy evidence at pace with the innovation and implementation needs of continuously learning health systems.

## Introduction

Delivering high-value care as a continuously learning health system requires aligning the rapid pace of change in evidence and practice with continuous improvement and innovation [1]. However, systems learning through the typical feedback loop of research and evaluation can fall behind fast-paced innovation cycles [2–4]. The use of rigorous, time-consuming experimental or quasi-experimental research and evaluation designs to assess an innovation’s impact is limited by deployment into clinical environments that operate on short, “business-cycle” timelines. Innovations tend to be complex, incorporating a number of interacting components that target multiple disciplines or organizational levels [5]. Such interventions are highly sensitive to contextual factors, and generally undergo significant adaptations during the implementation process, yet such depth of implementation understanding is often absent in evaluation [2, 3, 6, 7]. The wide range of challenges necessitates creative evaluation designs that produce rigorous, timely information that is critical to the adoption, adaptation, and implementation of effective innovations for the learning healthcare system.

Rapid assessment procedures (RAP) are a useful approach for producing contextually rich evaluative information on short timelines [8]. RAP describe a group of methods that have roots in rural development, real-time evaluation of humanitarian crisis, and public health in situations where quick, accurate, and actionable information is needed [9–11]. RAP typically has five common core features:

1. Use of mixed methods, typically with qualitative data collected through key informant interviews, focus groups, document review, and naturalistic observations; and quantitative outcomes or process data collected through reviews of documentation for secondary analysis and/or surveys;
2. Rapid timeline of weeks to months from start to finish;
3. Participation by the population of interest in planning and implementing the research;
4. Team approach to the research process; and
5. An iterative cycle of data collection and analysis [12].

RAP is a time efficient approach useful for studying naturalistic settings and their processes, such as organizational practices and implementation, and uncovering “how” and “why” things work [8, 13]. As such, the method has promise for evaluating quality and safety interventions in healthcare [13–15]. However, though RAP studies condense time in the field gathering data from months or years to days or weeks, the qualitative analysis process at the heart of RAP has not been substantially shortened [11, 16, 17]. To compensate, studies have been designed around transcription timelines, which can take several weeks, and strategies have been used to divide researcher time to limited aspects of analysis [17], yet these do not represent substantive improvements to the analytic journey.

This paper describes a RAP approach incorporating implementation science frameworks and a time-sensitive team-based qualitative analysis process to enhance the speed with which RAP studies can be completed. We modified RAP in response to the challenges of evaluating multiple complex programs in evolving healthcare settings on a short timeline, while at the same time generating evidence informed by implementation science needed for health systems learning. We illustrate this approach using a case example of an evaluation to assess implementation of a complex program to improve patient safety in intensive care units (ICUs) in four academic medical centers (sites). Our aim for evaluating each of the four academic medical center sites was to assess the implementation of the set of innovations/interventions implemented. Specifically, we aimed to identify why and how the package of interventions was developed and implemented; the unit, organization, and regional context for implementation; and barriers and facilitators faced during implementation for each site.

## Methods

### Design

Our RAP approach combined elements from a number of “rapid” approaches (rapid appraisal, rapid evaluation, rapid assessment) [12]. We drew on McMullen and colleagues’ [13] critical elements in the rapid assessment process and tailored our approach by incorporating established implementation science frameworks into data collection and analysis. We also developed a team analysis process to further speed the evaluation timeline to ensure we could evaluate and draw comparisons across four distinct site projects and deliver a report to stakeholders within 6 months. Our design process was guided by Miles and colleagues’ quality standards for qualitative research and the Standards for Reporting Qualitative Research were utilized for reporting [18, 19]. This study received a non-research determination from the Institutional Review Board because it evaluated quality improvement programs.

### Setting and interventions

We assessed a portfolio of patient safety and quality innovations deployed in four academic medical center sites in the USA between May and November 2016. Multidisciplinary teams in each medical center received grants to redesign ICU care with goals of reducing adverse hospital-acquired events, improving patient and family engagement, and reducing costs in the ICU. All four medical center sites developed a unique suite of interventions with each of the following three features:

1. Electronic patient information/communication portals to allow patients and family members to engage in the ICU care process;
2. Interactive information technology (IT) tools for use by care teams to aggregate, display, and respond to the status of key safety practices (such as deep venous thrombosis prophylaxis, or delirium assessment); and
3. Interventions to improve culture, provider behavior, or workflow around patient safety or communication.

The projects were in various stages of implementation at the time of our evaluation and many of the innovations had been implemented in a quality improvement framework. The site implementation teams, comprised of clinicians and academicians, had performed or were currently performing internal effectiveness evaluations of their projects with pre-post designs. Findings from the effectiveness evaluations were reported by sites and included as part of our document review; we did not conduct our own effectiveness evaluation and do not present their data here due to data sharing restrictions.

### Data collection

The steps in our process are described in Table 1 and steps 4–7 were repeated for each of the four sites. We launched our process by working with the program funder to agree on the evaluation questions and goals, a key step to ensure that the evaluation products aligned with their information needs. Consistent with program evaluation best practices, we then developed and refined a logic model based on the program theory of change in collaboration with the funder to focus and guide data collection [5]. Pinpointing the focus of the study is an essential element of rapid qualitative approaches [20].

**Table 1:** Steps in the rapid assessment procedure process

Stage	Step
Design	1. Iteratively worked with the program funder starting with kickoff meeting to establish program logic model, focus questions, and ensure evaluation products met stakeholder needs.
	2. Incorporated implementation science frameworks *a priori* in the development of the field guide and analytic tools.
	3. Selected the site visit team (3–4 researchers) to have varied methodological expertise and content knowledge. Carried out group training to align data collection techniques and practice using the field guide.
Data collection and analysis	4. Established rapport with site liaison via preparatory phone calls to gather background information. Gathered and analyzed existing datasets from site’s internal pre-post effectiveness evaluation and quality improvement projects in preparation for site visit. Carried out secondary analysis of site outcomes.
	5. Visited site over 2 days for qualitative data collection. Visit began with presentation by site team, followed by formal and informal interviews, meetings, observations, and demonstrations in the ICU.
	6. Through team discussion, sifted qualitative data from one source (e.g., interview/observation/meeting) into a chart developed from implementation frameworks. At the end of each day and on return to the office, triangulated data from the charts to develop themes.
Validation and reporting	7. Findings from each site visit were written up as a case summary in the two weeks following the visit. The first stage of the writing process was carried out by the qualitative lead (LMH) and was part of the analytic journey as findings from the secondary quantitative data analysis from the document review were written up alongside site visit findings. Any points of inconsistency or which needed clarification were discussed by the research team as a whole until consensus was achieved. The site summary was shared with the site for validation.

Data collection through multiple modalities is a key feature of RAP and structured field guides facilitate focused data collection and analysis [14]. Our field guide, outlined in Table 2 and modeled on McMullen et al.’s tool [13], contained logistical information, sets of interview topic guides, a structured observation form, field survey instruments, and analytic tools for rapidly sifting data into predefined categories of interest through team discussion. Our field guide included a summary of findings generated from site documents, including their internal effectiveness/quality improvement evaluations so that we could explore with sites how and why reported outcomes were achieved. All documentation was contained in a single binder, one for each researcher per site, where all individual notes and data were recorded and organized. Our interview and observation guides were structured to understand the context and process for implementation, drawing from both the Reach, Effectiveness, Adoption, Implementation and Maintenance (RE-AIM) framework [21] and the Consolidated Framework for Implementation Research (CFIR) [22], both well-established frameworks with compatible concepts for collecting and analyzing data about implementation [23]. We chose to structure our tools around these established frameworks to help improve consistency in our process because they have well-defined, distinct conceptual definitions relevant to our evaluation questions and were applicable to the broad scope of each site’s implementation.

**Table 2:** Contents of field guide

Document	Description
Site visit plan	Plan of work for each day of an average 2-day site visit.
Site schedule	Provided by site.
Site summary and results	Overview of site projects including intervention descriptions, interim and final reports, internal evaluation/quality improvement findings, and quantitative data on process and outcome measures (generally pre-post data) which we analyzed to standardize outcomes and findings across the four sites.
Informant list	List of key informants and people involved in the site project, including list of people to be interviewed.
Questions for site team during Q&A	Questions for clarification posed to the site team to answer during first half day which typically included presentations.
Focus questions	The main questions that the evaluation sought to address.
Hospital site profile instrument	Profile initially completed by evaluation team which gave a contextual overview of that site. Given to the site lead during the visit for completion and accuracy check.
Interview guides	Topic and question guide for: site principal investigators and co-investigators, project managers, clinician leaders, administrative leaders, implementers, chief quality officers, patient, and family advisers. Questions were driven by CFIR and RE-AIM concepts and included the following topics: • Background questions about the person’s role in the project; • What interventions were implemented and why; • How implementation of the project went, including: whether it went as planned; adoption and adaptation of the interventions; barriers and facilitators; influence of organizational culture and infrastructure; resources needed for implementation; and potential for maintenance of the interventions; and • Spread and scalability of the project within and outside the organization.
Field survey form	Administered to ICU staff/providers about their experience of using the interventions during observational periods. Contained structured questions and open-ended questions to be used as suitable.
Field note form	Observational notes were recorded on blank paper and notes summarized using the field note form.
Implementation analysis chart	For team summarization of findings and interpretations from interviews or observations in a structured way to facilitate further analysis.
CFIR constructs and RE-AIM framework	Printouts of the frameworks and construct definitions as a reference sheet.

Our site visit team included a multidisciplinary group of three health service and implementation science researchers to provide a diversity of perspectives and reduce individual biases [13]: a doctoral trained qualitative researcher as there is heavy reliance on qualitative methods [9, 10], an internal medicine physician as innovations were highly medicalized, and masters-level project manager with a public health background. On two visits, an epidemiologist leading the secondary analysis of quantitative data as part of the document review joined the site visit team to clarify the data from the internal evaluations and to gain contextual insight. A common criticism of the qualitative paradigm relates to ensuring freedom from bias and the objectivity of the study [24]. We accepted and made explicit the perspectives of the researchers with stakeholders so they understood the representation of “truth” in the findings and could assess their credibility [18]. Careful selection of a multi-disciplinary evaluation team was essential as the quality of the work depended on a group process of data collection, analysis, and interpretation, and also added a dimension of triangulation among researchers [8]. Prior to starting the site visits, the core team of three researchers trained together over a half day to practice using the field guide and align data collection techniques, such as what notes to take during observations and how to record informal data.

We worked with one site liaison (usually the project director) from each site before, during, and after the site visits to identify and access the right people to interview, and locations or events that should be observed. We shared the evaluation questions and our scope of work so that the aims and primary evaluation questions were transparent to the sites to establish rapport and build trust with site teams, which was necessary for a participatory approach. Building rapport is an essential element in ethnographically founded methods which rely on close, open contact with subjects and settings of interest [25].

Visits began with site teams presenting their projects and introducing their teams, followed by multiple rounds of formal interviews and observations punctuated by informal discussions. Interviews and group discussions were recorded for reference, and one researcher took detailed notes on a laptop. Our team worked together during site visits, usually with at least two team members carrying out observations or participating in interviews. The team convened throughout the day between all formal data collection periods to reflect on initial impressions, retune interview questions, and identify additional opportunities for data collection and points for clarification. This team approach involved continual dialogue between researchers and reflection helped to assure consistency, validity, and completeness in data collection [18]. In addition to our team approach to data collection, site visits proceeded with close, iterative discussion with key individuals at each site who were usually the site liaison and principal investigators. Besides the interviews and observations, there were frequent informal opportunities to explore our understanding with key informants in a transparent, participatory fashion [26]. We frequently drew on realist interview techniques in order to confirm or disconfirm our understanding of their experience [27]. This meant that our conclusions reflected both our interpretation as external evaluators and the perspective of the key individuals who were closest to the work when it was happening.

With the site visits, and indeed with qualitative methodology in general, a common question is “how much is enough?” Commonly, researchers approach reliability in terms of achieving thematic saturation, and studies have demonstrated that saturation can be achieved in as few as eight interviews or three focus groups [28], with more required to understand the full meaning of themes uncovered [29]. Data is generated using multiple methods, drawing particularly on methods rooted in anthropology [8, 9], and from multiple sources, from formally organized interviews with key informants, to opportunistic data that arose in hallway conversations walking between interviews. Additionally, our iterative, field-based team analysis process meant that we could incorporate in real-time additional questions needed to fully understand emerging themes within our predefined implementation concepts. The triangulation of data, methods, and sources contributes to an overall picture-building, therefore, specifying “how much” data is needed is not as relevant as the power of the information generated [30]. Malterud and colleagues argue that smaller samples will likely be sufficient if aims are narrow, experiences of interest are dense within the target population, evaluation is theoretically driven, and researchers are experienced in the subject matter [30]. The extensive preparatory work prior to and during the site visit, and in the follow up phone calls and emails all contributed to the bolus of information for enhancing the reliability of the study.

### Rapid team-based analysis using implementation frameworks

To simplify and thus speed our analysis process, we used a templated approach by creating analysis charts using a priori themes derived from concepts from RE-AIM and CFIR frameworks [8, 31]. RE-AIM conceptualizes the real-world process of translating research into action [21], whereas CFIR specifies constructs associated with effective implementation [22]. These frameworks are well-defined and formed a structured codebook which we organized as a chart into which we sifted data [32]. Evaluation-specific categories were added to the charts along with a category to capture emergent themes. Analysis began during the site visit during breaks in data collection and more formal analysis took place at the end of each day through group discussion. For interviews where detailed notes had been taken, the notetaker read the notes to the team, stopping to clarify any points and referring to recordings as necessary. For observations, each observer verbally summarized their notes with team members asking questions and prompting the observer to think more critically about their observations and assumptions [18]. Following these presentations of the data and using the thematic chart and CFIR/RE-AIM frameworks as a guide, each team member verbally reflected on what they felt the data raised about the thematic categories; e.g., what features of the inner setting (CFIR construct) influenced implementation or were notable. One team member wrote the groups’ consensus points in the chart coding them with the constructs from the implementation frameworks, thus condensing and clustering data within the predetermined themes [18]. Each data source (e.g., interview, observation) had one complete chart with summarized findings that had been discussed and agreed by the group.

We met with the site principal investigator and/or key members of the project team for the last meeting of the site visit. Prior to that meeting, our team gathered to discuss a summary of the visit and initial conclusions regarding each of the evaluation focus questions, being sure to note any questions or issues needing discussion. One researcher fed back our key takeaways and findings from the site visit as a validation check, similar to member-checking [18]. The final validation meeting proved a useful step in multiple ways: there were often points needing clarification that we could further discuss, it reassured the site project team that our findings represented their experiences, and gave sites the opportunity to help interpret findings as part of a participatory approach [9].

In the week following the site visits and our return to the office, the qualitative team lead (LMH) looked across the charts and documents collected from the sites to synthesize findings across sources by themes. Notes were written into a new chart, with any outlying data noted for discussion with the group. The synthesized findings were then discussed again by the team until a consensus of the findings was reached. The synthesis was then presented to the wider evaluation team, which included two health services researcher physicians, an epidemiologist, and an ICU nurse. At this point (the interpretation stage), qualitative findings were triangulated with the secondary analysis of quantitative data [33]. We used discussion among the wider evaluation group to test the strength of evidence gathered during the site visits as we examined the various datasets for corroboration or divergence in findings between sources and methods [33]. The wider group did not have firsthand depth of knowledge of the qualitative site visit data to assert a different interpretation, but rather they could posit a rival hypothesis for the smaller group to consider which then had to be checked against the data to either be refuted or confirmed, and then the interpretation amended as needed. Whereas traditional qualitative analysis methods are increasingly computer-based using software [34], our approach was primarily verbal and paper-based with much of the analytical thought process happening in a group discussion and funneling the data using implementation frameworks to distill key findings regarding focused evaluation questions [8].

### Validated findings with site teams

In the weeks following each site visit, we wrote a site case summary while the experience and data were fresh in our minds. Using the final synthesized chart, one researcher took the lead to write the findings, which helped to crystalize our analysis and interpretation. The draft was circulated among the wider team and iterated on until it reflected the team’s understanding of the data. The summaries were then shared with the site project teams as an additional validation check to ensure that our assessment of their implementation resonated with their lived experience. Any inconsistencies between site visit data and documents collected from sites were highlighted to the sites for clarification. This process of checking and re-checking findings with the sites enhanced the authenticity and credibility of our accounts of the implementers’ experiences [18]. Our summaries helped to clarify our own interpretations, but also proved to be useful learning for sites in mid-implementation as our outside perspective served as a snapshot of progress within their own context of ongoing change. As is the norm in qualitative research, we were not aiming for generalizable data, but rather sought to understand events within a particular context and use thick description to enhance transferability [18, 35]. While generalizability may be limited, this approach aimed at producing actionable findings for stakeholders, which included the funder and the healthcare systems.

## Results

We repeated the method described above for each of the four site evaluations. Table 3 summarizes the data collected at each site, the interventions implemented, and the site’s experience of implementation summarized as barriers and facilitators. Below we present a more detailed summary of findings from site D to illustrate the results from our application of the RAP approach. The results presented here reflect primarily qualitative data generated from interviews and observations during site visits and gathered through document review as part of the RAP approach. We do not present quantitative effectiveness/quality improvement and implementation data from the site’s internal evaluation reports due to data sharing restrictions with the site.

**Table 3:** Data collected, interventions implementation, and facilitators and barriers to implementation by site

	Site A^a^	Site B	Site C	Site D
Data collection^b^				
Documents	26	17	13	42
One-to-one interviews	–	3	10	2
Group interviews	5	2	–	5
Observations, including informal conversations	–	2	2	3
Field survey	–	2	–	–
Presentations	5	2	3	2
Demonstrations of innovations	–	1	1	1
Interventions implemented				
Electronic patient information/communication portals	Portal with information about the ICU, care team, and option for patient/family to upload information about themselves for viewing by care team. Accessed via bedside iPads.	Portal with information about the ICU, care team, and option for patient/family to upload information about themselves for viewing by care team. Accessed via bedside iPads.	Portal for communication with care team, care plan information, and educational tools. Accessed via bedside iPads.	Portal to enhance patient/family engagement and educate patients/families about care in the ICU. Version 1 accessed via bedside iPads; version 2 via any personal device.
Interactive provider IT tools	Care team portal to display harms status at ICU level; Failed implementation: sensors to integrate ICU devices with care team portal.	Care team portal to display harms status at ICU level.	Tool included: care plan summary, nursing care plan, safety checklist, and communication with patients/families and other providers.	In development: Predictive algorithm for identifying harm; electronic patient safety checklist.
Interventions to improve unit culture, provider behavior, and/or workflow	Standardized program to escalate safety issues to management.	Standardized program to escalate safety issues to management.	Structured, paper-based tool for guiding communication with patients/families.	Redesigned rounds to include nurses; standardization of room entry; standardization of policies and practices.
Facilitators and barriers to implementing the interventions				
Implementation facilitators	History and prior experience within the unit with research and innovation in patient safety meant clinicians were willing to implement changes. Transdisciplinary implementation team utilizing skills and expertise from a wide range of people working as one, integrated group. Co-location of key project personnel from different disciplines enabled transfer of important innovation development techniques and information. Used an innovation prototype for clinicians for test outside of the ICU.	Interprofessional work culture enabled equitable participation of different professional groups (e.g., doctors, nurses, pharmacists, physical therapists) in innovation development and implementation. Belief among clinicians in the value of the provider IT tools for creating situational awareness needed to reduce harms in the ICU. Clinical “super-users” of the provider IT tool to support adoption and use among clinicians in the ICU. Site B was able to work with Site A to learn from their implementation experiences prior to implementing in their organization. The “zero harms” goal of the project was aligned with the institutional priorities which helped to increase adoption and potential for maintenance of changes.	Strong medical and nursing leadership provided top-down support for implementing changes. Development of innovations followed observations of workflows to improve integration into existing workflows. Existing checklist culture in the ICU made adoption of an electronic patient safety checklist easier. Previous team experience developing and implementing IT innovations, and established relationships with enterprise IT developers. Patient portal was initially tested as a prototype in a separate phase for refinement prior to implementation which allowed an improved tool to be implemented.	Patient engagement culture embedded at all levels of the organization led to easy acceptance of patient engagement efforts among clinicians. Engaged frontline staff in innovation design across all ICUs to ensure integration with unit-specific workflows. Common governance structure and alignment of processes across critical care led to consistent adoption. Acceptance and embrace of innovation development and implementation as a learning process.
Implementation barriers	Lack of application program interface (API) for integrating provider IT tools with medical devices to produce a “smart” ICU. Lack of fit of prototype into real-world clinician workflows. Lack of clinician readiness for workflow changes. IT glitches from enterprise clinical system updates corrupted the outputs from the provider IT tools. Lack of relationship with enterprise IT needed to integrate IT innovations with the EHR. Mismatch in timescales to achieve the scope of vision for change and produce outcomes resulting from that change. Building relationships among experts who had not worked together before took time to develop. Complex IRB consent processes for patients with high acuity reduced patient portal adoption.	Cost of implementing and maintaining the IT interventions was high and may prohibit spread across the organization. IT glitches (e.g., loss of connectivity to WiFi, software updates, etc.) slowed uptake due to poor user experience. Cycle time for adapting the IT interventions’ software for further refinement was felt to be too slow and expensive. Mismatch in timescales to achieve the scope of vision for change and produce outcomes resulting from that change.	Established methods of communication and workflow used by providers led to low adoption of provider IT communication tool. Open unit configuration in which physicians rotated in and out meant it was hard to get provider adoption of IT communication tools. Instability and turnover in the workforce were disruptive to the unit. Static, unit-based hardware devices limited access by patients/families to the patient portal. Complex IRB consent processes for patients with high acuity reduced patient portal adoption.	Lack of alignment between timeframe associated with project grant and expectations for health service innovation to produce measurable impact. Regulation of protected health information limited accessibility of patient portal.

^a^Site A was implementing a new electronic health record at the time of the visit and therefore no interventions were operational for observing.

^b^Data sources varied by site as specific features of the three categories of interventions across sites varied and reflect the availability of implementers/personnel at the time of the site visit

Site D implemented a complex package of innovations across eight ICUS: a redesigned rounding process to more fully involve nurses, a standardized room entry procedure to decrease infection risk, standardization of policies and procedures across all ICUs to align practices, and a patient and provider communication portal. Several of the innovations were still being developed or were being adapted at the time of our visit in August 2016, including a predictive model to identify increased risk of harm, a provider facing electronic patient safety checklist, and a second version of the patient and provider communication portal. The findings presented here focus on the innovations that had been implemented at the time of the site visit.

### Approach to innovation development and implementation

The overall experience of implementation was characterized by a culture of work that highly values the role of nurses and input from patients and family and this was reflected in how the innovations were developed and implemented. In particular, standardizing room entry, rounds redesign, and the patient portal were reflective of the concerns of patients and families regarding hand hygiene and informational needs, and the desire to ensure full representation by nurses in care decision making.

The site’s approach to implementation was underpinned by a quality improvement culture which appeared to be a strength in their implementation process. By viewing the development of the innovations as a learning process, they were not seen to be tied to a project timescale, but rather the focus was on developing sustainable innovations and change of practice.

### Interventions to change provider behavior and workflow

Redesigning rounds was initiated to increase the inclusion of non-physician clinicians on rounds to encourage open communication in patient care decision making. The site implementation team spent 6 months collecting data, developing, and testing the rounding intervention with a group of frontline staff from all units before rolling out across all ICUs. The intervention was intentionally designed to be simple and flexible enough to be adapted to unit-specific workflow. We observed that the redesigned process was embedded in the rounds workflow in the two ICUs we visited and that the nurses, in particular, viewed this as a highly successful program to enhance nursing participation in rounds:

It gives you a platform to talk about what’s at the forefront of the nurse’s mind that may not be at the forefront of the rest of the team’s mind. (Site D, Interview 05)

Standardization of the steps for room entry was driven by a perception that hand hygiene was an “industrial-grade” process, happening thousands of times a day. As the project evolved, the project team learned through the patient and family advisory council that, in addition to hygiene, patients also had concerns about whether and how clinicians introduced themselves upon room entry, and expressed distress about instances where clinicians would physically touch them for clinical care without asking permission. This feedback from patients became a strong driver for the project team:

The voice of patients and families have been screaming the past two years: “This is what it feels like when an attending switches to a new attending”; “This is what it feels like when you enter my room at night and touch me” (Site D, Presentation 01)

Redesigning the room entry process involved the creation of a standard, multi-step process and a cart to be placed within rooms for performing hand hygiene and gowning, as necessary, in full view of the patient. However, mid-project organizational level changes in gowning requirements for infections meant that, over time, the cart was used primarily for hand hygiene and was more of an encumbrance, particularly in older rooms which lacked square footage. Also, staff were required to identify themselves each time they entered the room and explain what they were doing. The observational evaluation carried out by the site implementation team showed mixed adherence to the standard entry process, with nurses seemingly having greatest compliance and doctors the poorest. During the site visit, it was observed on one occasion that a nurse entered the room to check the monitor screen and left within 5 s, and though she sanitized her hands, she did not say who she was or what she was doing there to the patient who was unconscious (observation 02). Reflecting on the site documentation, our observations, and feedback from staff, it seemed that the process for room entry was not consistently adopted, perhaps because it was not always feasible to implement, and was not sustained over time.

### Electronic patient portal

The site implementation team reported that an initial pilot of the electronic patient portal in two ICUs had low overall uptake of 14% (49 patients of 352 admissions). Due to the poor health of the patients, the portal was predominantly used by families. After obtaining feedback from clinicians and patient/family users, the implementation team adapted the portal and launched a second version in July 2016 across all eight ICUs. The new version retained the portal’s focus on providing patient/family users with information about day-to-day processes in the ICU, but made several modifications to the user interface. Version 2 was designed to be mobile friendly so it could be accessed on any device and the information contained was restructured so that users were presented with only the information they were interested in. The new version was also stripped of protected health information (PHI), allowing it to be accessible on personal mobile devices without additional security requirements:

This [patient portal] is devoid of PHI which does not require consent or create privacy issues with log-ons. There are some limitations related to this. [This is different to Site C which] has more clinical content and have people consent and have paid a price in the number of people they are able to consent. (Site D, Observation 01)

### Facilitators of implementation

#### Patient engagement culture embedded at all levels

A consistent theme across all interviews and observations was how valued and central the patient voice was to the organization. Patients and families were not a group from which advice on a topic was sought, but rather a voice that drove what topics were discussed. Therefore, for interventions such as the patient portal which aimed to engage patients and families in their care, support from clinicians for their patients to use the portal almost seemed to come naturally as clinicians felt the portal was an extension of their philosophy of valuing patient participation.

I do think here providers, it’s a little bit different and I do think that because patient family engagement has been around here for a long time they didn’t need to buy into it so much [...] in terms of engagement, there was already a buy in (Site D, Interview 03)

#### Engaged frontline staff in innovation design across all adopting units

Teams from all units were involved in the design process for both rounds redesign and standardizing room entry. The teams agreed on a set of core elements that were simple, yet flexible enough to be adapted to differences in unit workflow. Staff engagement was particularly successful for initial adoption, especially for rounds redesign, where each of the eight ICUs had implemented the rounding process and, as a result, reliably incorporated the nurse’s voice for most patients each day.

#### Common governance structure across critical care

Within the institution, there was one common governance structure across all ICUs, and alignment of key practices and processes in all units. This set-up enabled the innovations to scale to all units.

#### Acceptance of innovation development and implementation as a learning process

The process of innovation development and implementation was expected to be iterative and thus paced at a speed to allow for learnings to be incorporated into design and implementation. There was acceptance that a lack of experience in IT design for the patient portal required extra time to learn. Rather than this putting pressure on the implementation team, any potential problems were viewed as a learning opportunity which could be incorporated into the implementation process.

### Barriers to implementation

#### Lack of alignment between business cycle timeframe and health service innovation

It was felt that the 3-year timeframe associated with the project grant was not practical for health service/technology innovation and implementation into a dynamic clinical environment. The project team considered the funding period to be too short to deliver measureable clinical outcomes, especially when innovations were starting from scratch.

The expectation that you'd be able to get something dramatic signed, sealed, and delivered at three years isn't realistic. We're at a point now [3 years after the start] where people are hitting their stride and have the ground work done and are ready to take opportunities to spread. (Site D, Interview 02)

#### Regulation of protected health information

There were strict rules around how PHI could be accessed and how users had to be consented for testing of version 1 of the patient portal. As a result, the first version was implemented on ICU-based iPads, which limited acceptability and adoption by patients and families.

## Discussion

We described an adapted version of RAP methodology using implementation science frameworks and a team-based approach to rapid analysis and synthesis that can be done in the field, thus saving days and weeks of costly transcription and analysis time. Our evaluation yielded important findings about the implementation of each of the four site’s projects, such as barriers and facilitators to implementation and organizational and cultural influences on the process. We found that using a modified version of RAP produced contextually-rich information using robust data collection methods within a short timeframe. There is value in this approach for evaluating quality and safety initiatives in healthcare, particularly for the learning health system, as it engages stakeholders in digging deeper to uncover new insights about known issues while stimulating learning for those involved [36]. Drawing on quantitative data from secondary sources (e.g., as part of a document review) and primary qualitative data offers a robust design in the field of patient safety which traditionally has lacked mixed methods approaches [37]. Other methods, such as a survey or analysis of secondary data alone, may not have produced the depth of understanding of barriers and facilitators to innovation, implementation, and measurement, nor generated lessons with the level of granularity needed to interpret the findings across these complex quality improvement programs.

While rapid assessment processes can speed up the data collection phase of evaluation processes, they are still typically plagued by traditional, slow analysis methods [38]. There have been a variety of attempts to improve the speed at which rigorous qualitative analysis can be undertaken. Such approaches include creating an a priori structured codebook for summarizing data by interview question [39], coding audio only [20], condensing data down through a series of tables [40], or allocating researchers to code for specific themes [17]. Such rapid analysis approaches have been shown to produce valid findings compared to traditional in-depth, line-by-line transcript analysis and may be added to the applied qualitative health services researcher toolkit [39]. However, they largely still rely on the production of transcripts and office- or computer-based work. Our study contributes a rapid approach to team analysis that has thus far been lacking for RAP [41]. Our approach is similar to other templated rapid methods as it requires a structured approach to evaluation questions and analysis [8], but the team analysis process shortens analysis time by creating an iterative group process of reviewing and reflecting on the data, sorting and categorizing, and meaning-making using charts, and does not require transcripts. We do not minimize the value of transcripts, as a close reading of detailed transcripts is certainly worthwhile for many, if not most, research questions. But data can take different forms and where questions are focused with a limited number of pre-defined categories and the time gap between data collection and formal analysis can be minimized, this is an approach which retains features important to achieving high-quality research, yet can be carried out quickly. It may miss out on identifying potentially interesting, unexpected topics, but that is a trade-off for producing quick information on pre-specified categories.

Success for rapid team-based analysis hinges on a number of factors: structured field guide, careful selection of the site visit team, clear analytic purpose, space and time during the site visit for team discussion and charting, and a validation process with member-checks. The team dynamic is the focal point of this process and it is important that the same team members participate if data is collected iteratively since knowledge and understanding accumulates as data collection progresses. Threats of bias in field notes from researcher interpretations are minimized by having multiple researchers present who are willing and able to challenge each other’s ideas and beliefs about the data [42]. Though the goal of team-based coding is to apply codes consistently across the dataset, this is usually done independently and then compared, thus enhancing validity and reliability [43]. Our approach using discussion puts consensus formation upfront, so it is essential that researchers state if they disagree and thus may not be suitable among teams where members perceive a power imbalance. This approach only reflects a team analysis if the whole team is fully engaged as equal members.

Documentation is a challenge for this approach which limits the auditability of the process as the typical coding process is truncated into a verbal discussion [18]. In the future, it would be advisable to record the group discussions in which we completed our data charts to provide an auditable trail [43]. Admittedly, the rapidity of our analysis meant that we did not progress into higher levels of abstraction and interpretation, but rather focused on recurring patterns, themes, and clustering of data bounded by the implementation frameworks. Indeed, the analytic speed as a team was achieved by using well-defined, structured implementation frameworks familiar to the team which was appropriate given our aims. However, a drawback to this approach is losing the “voice” of the data and thus not likely appropriate for exploratory research questions [32]. This methodology is not limited to the use of these specific implementation frameworks, but rather researchers should select a theory or framework that is most appropriate to their research question. Future research might look at comparing findings from a rapid team-based analysis to standard content analysis approaches.

## Conclusions

RAP is suitable when contextually rich, evaluative information is needed quickly. Inclusion of implementation science frameworks allowed us to generate evidence about implementation process and outcomes associated with effectiveness and underpinned our team-based analysis process. The features of RAP methodology with rapid team-based analysis that are particularly suitable for studying implementation retrospectively with limited time include:

- Focus on a participatory approach in which participants not only provide data but help to inform how that data might be interpreted into action and validate conclusions;
- Rapid, multidisciplinary team-based approach to analysis improving speed while ensuring trustworthy, credible findings;
- Emphasis on process and operations; and
- Reliance on qualitative methods to gain insight into actions located within a particular context, giving high internal validity.

A team-based approach to analysis utilizing implementation science frameworks can further improve the pace at which RAP studies can be conducted thus getting important, credible lessons about implementation into practice sooner.

## Supplementary information

**Additional file 1.** Standards for Reporting Qualitative Research checklist.

## Data Availability

The datasets generated and/or analyzed during the current study are not publicly available due to privacy concerns, but are available from the corresponding author on reasonable request.

## Abbreviations

API: Application program interface
CFIR: Consolidated Framework for Implementation Research
EHR: Electronic health record
ICU: Intensive care unit
IRB: Institutional review board
IT: Information technology
PHI: Protected health information
RAP: Rapid assessment procedures
RE-AIM: Reach, Effectiveness, Adoption, Implementation and Maintenance
